# An innovative transactive energy architecture for community microgrids in modern multi-carrier energy networks: a Chicago case study

**DOI:** 10.1038/s41598-023-28563-7

**Published:** 2023-01-27

**Authors:** Mohammadreza Daneshvar, Behnam Mohammadi-Ivatloo, Kazem Zare

**Affiliations:** 1 Faculty of Electrical and Computer Engineering, University of Tabriz, Tabriz, Iran; 2 Department of Electrical Engineering, School of Energy Systems, Lappeenranta University of Technology, Lappeenranta, Finland

**Keywords:** Energy economics, Energy efficiency, Energy management, Energy supply and demand, Hydrogen energy, Solar energy, Wind energy, Batteries, Hydrogen storage, Energy grids and networks, Power distribution

## Abstract

As the technology of multi-energy carbon-free systems is strikingly developed, renewable-based multi-vector energy integration has become a prevalent trend in the decarbonization procedure of multi-carrier energy networks (MCENs). This paper proposes a fair transactive energy model for structuring an innovative local multi-energy trading market to allow multi-carrier multi-microgrids (MCMGs) with 100% renewable energy sources (RESs) in Chicago for free energy exchange aiming to balance energy in the renewable-dominant environment. Indeed, the main goal of the proposed model is to facilitate the modernization of future MCENs that are targeted to be equipped with 100% RESs and require a holistic model engaged with innovative technologies for the realization. To this end, the transactive energy architecture is designed for techno-environmental-economic assessing hybrid MCMGs to increase their flexibility in unbroken energy serving, decreasing their dependency on the main grid, and improving their economic benefits by considering their contribution level in energy interactions. To effectively model uncertainties of MCENs with 100% RESs, the novel hybrid technique is proposed that considers various stochastic changes of uncertain parameters to achieve confident results. The results highlighted the capability of the proposed model in effectively utilizing fully produced clean energy as well as continuously multi-energy serving of MCMGs in the presence of 100% RESs. Moreover, MCMGs reached techno-environmental-economic benefits by operating under the proposed transactive energy-based model, in which the technical, environmental, and economic goals are respectively realized by considering all constraints of MCENs, producing 100% clean energy by RESs, and reducing the total energy cost from $1,274,742.55 in the based model to $1,159,235.89 in the proposed one.

## Introduction

### Motivation and background

NOWADAYS, energy growth in its diverse types is accompanied by tremendous advances in technologies for multi-energy systems that welcome hybrid energy networks more than ever before. Under this trend, co- and tri-generation units have widely emerged and attracted remarkable attention from both industry and academia that is resulted in tightening the linkages between the electric power system (EPS), natural gas grid (NGG), and district heating network (DHN)^1^. In parallel with evolutions in the multi-carrier energy systems, economic and environmental concerns regarding fossil fuel-based energy units have driven the system to substitute them with renewable energy sources (RESs)^2^. Indeed, significant developments in multi-energy carbon-free technologies and diverse renewable systems, along with a diminution of the carbon-intensive end-user parts, have made the energy landscape experience accelerating change. In this regard, ambitious attempts are forming for successful transiting from the independent centralized energy grids towards the interdependent modern multi-vector energy grids with 100% RESs that are recognized as the path to grid modernization^3^. Indeed, future modern energy networks not only need to be a couple of multi-carrier energy networks (MCENs) for effectively using their interactions due to the rapid proliferation of hybrid energy systems but also are targeted to properly use the advantages of the environmental potential for fully unpolluted multi-energy production by operating 100% RESs^4^. Herein, two key questions can be raised as critical challenges. The first one is how can several energy networks such as EPS, NGG, and DHN be effectively operated in the presence of 100% RESs. In other words, due to the existence of hybrid energy systems throughout the integrated structure, the dependency among the EPS, NGG, and DHN is tight, and modeling their interactions in the interconnected mode is necessary for reaching confident results. The second question is about the innovative ways and technologies for ensuring uninterrupted energy meeting in the sharp deregulated area with fully intermittent energy producers. Indeed, the incorporated system with 100% RESs requires innovative ways and technologies that not only can increase the reliability of supplying energy in the presence of full renewables but also can improve the interoperability between the variety of components in the MCENs infrastructure. Thus, the great requirement is felt for the plenary model that plays a crucial role as strong leverage in effectively interconnecting different energy networks by especially improving the interoperability among them while integrating numerous RESs for fully pollutant-free multi-energy production in a reliable and sustainable way. To this end, this paper is aimed at proposing a holistic model for optimal scheduling of multi-carrier multi-microgrids (MCMGs) equipped with 100% RESs in the coupled structure of the EPS, NGG, and DHN.

### Relevant literature

In recent years, as the development of multi-energy systems along with their dependency on each other have substantially come into view, energy network infrastructures have undergone a progressively rapid change from traditional centralized systems to decentralized modern integrated networks. This transition has brought the idea of scrutinizing the already isolated structures of the EPS, NGG, and DHN in the whole interconnected infrastructure^5^. In the conventional system, fossil-fuel based energy production devices hinder the efficient, cost-effective, and clean operation of energy grids, which has been resulted in the inspiration of innovative rescheduling ideas regarding the substituting of them with RESs. In this respect, the booming deployment of carbon-free multi-energy facilities has led to the consensus in mushrooming cleaner energy mix generation devices with near-zero greenhouse gas emissions^6^. Albeit the operation of 100% RESs offers tremendous advantages in terms of economic and environmental, their unpredictability attributes in energy generation have faced the system with pivotal challenges. These challenges mostly stand on threatening the confident adjustment of a time-to-time energy equilibrium between the energy supply-side and demand sector. Thereby, developing innovative technologies and durable ways is crucial for the future modern grids with 100% RESs to allow the system to become more flexible in unremitting multi-energy supply. In this regard, energy transaction mechanisms are recognized as one of the reputed ways for balancing energy in the high renewable-penetrated grids^7^. Herein, transactive energy technology has received remarkable attention due to its capability in enabling the full renewable-penetrated system for sustainability^8,9^. Up till now, transactive energy has transpired as a potent contender in orchestrating the integrated operation of MCENs. In^10^, the authors proposed a two-level network-constrained model using transactive energy technology to enable multi-microgrids for peer-to-peer (P2P) energy transacting with the purpose of flexibility enhancement. Furthermore, the authors offered a nested transactive energy methodology in^11^ for effectively using the mutable attributes of residential consumers for increasing the flexibility of the demand-side. On the other hand, transactive energy is used in^12^ and^13^ to develop a novel blockchain-based paradigm to design a decentralized energy trading algorithm and promote economic and technical models.

In MCENs, effective integration of RESs can assist in tackling resource scarcity, profitable and affordable energy production, and alleviating environmental concerns^14^. However, large uncertainties come from their widespread diffusion and have challenged the reliability of the unbroken energy supplying, in which capable techniques are needed for realistic modeling of renewable-based systems^15^. Recently, several techniques are exerted for uncertainty management in different research works, including the stochastic programming (SP) method for optimal energy management of the industrial microgrid in^16^, robust optimization (RO) technique^17^ for the practical scheduling of microgrids in^18^, information gap decision theory (IGDT) for energy management of microgrids in^19^, chance-constrained programming (CCP) method for optimal gas-power flow problem in^20^, distributionally robust chance-constrained (DRCC) for the day ahead scheduling of microgrids in^21^, just to name a few. An exact evaluation of the aforementioned uncertainty modeling approaches resulted in the discovering of some remarkable drawbacks, which need to be intended in the energy grid modeling with 100% RESs. High complexity, lack of generality for modeling all types of uncertain parameters, time-consuming, and high computational burden are some of these important features. Indeed, because we witness the variety of uncertainties with different behaviors in both energy generation and demand-side, an appropriate technique needs to be exerted for effectively modeling these diverse fluctuations. For example, the DRCC methodology is more proper to model the intermittences of uncertain parameters that have deviations in small intervals such as energy price. This is because this method considers the worst state of uncertain parameters and using it for dealing with volatilities of uncertain parameters with variations in large intervals (such as wind speed) may lead to away-reality results. Indeed, when the DRCC intends the worst state of wind speed in the system analyzing, if the best state of wind speed occurs in real-time due to its uncertain nature, we will have a considerable difference among the scheduled results with the results obtained in the real-time given the existence of a large difference between the upper and lower levels of the uncertainty set. For this type of uncertain parameters (such as wind speed), using the SP-based methods will be useful because they model most of the occurrence state of uncertain parameters by generating scenarios and taking their occurrence probability into account in the uncertainty modeling process. In order to effectively use the advantages of the DRCC and SP techniques, this paper proposes a hybrid DRCC and SP technique for uncertainty quantification in the optimal scheduling of MCMGs.

### Research gaps and contributions

Given the investigated researches, several considerable gaps are detected as follows so that addressing them is crucial and inevitable for facilitating the modernization process of future MCENs.

1. As one of the prominent decarbonization steps of grid modernization, future MCENs are planned to fully utilize RESs for producing carbon-free energy in response to critical environmental and economic concerns of fossil fuel-based energy production units. Herein, one of the key challenges is how to develop a comprehensive structure to reliably facilitate the presence of 100% RESs with stochastic outputs as well as procure an appropriate condition for multi-energy interactions of MCMGs. As such a structure is a critical need for the promotion of the usage of RESs, the holistic and hybrid structure is not developed in recent literature for microgrids with multi-carrier energy and 100% RESs to not only support their active presence in MCENs’ interactions but also to fully utilize the different benefits of the system with 100% RESs.
2. The future MCENs with 100% RESs require different flexibility improvement technologies to alleviate the negative effects of stochastic fluctuations of RESs on continuous energy serving. Although employing energy-sharing technologies as one of the effective ways of flexibility enhancement is essential for ensuring a secure energy supply, the capable technology and an appropriate energy market structure are not offered in recent works for MCMGs to enable them for multi-energy trading aiming to gain different valuable achievements and facilitate the modernization process of MCENs.
3. In this research, as intended MCMGs are equipped with 100% RESs in their energy production sector for fully producing zero-emission multi-energy, uncertainties of various types of RESs are an inevitable part of the operation of the hybrid system. Herein, because of employing different sources of RESs, their multifarious stochastic behaviors in energy generation need to be effectively modeled. Previous studies ignored the development of an appropriate method to take various volatilities of RESs into account in the uncertainty quantification process. Indeed, an effective uncertainty modeling technique is not envisioned in recent studies to simultaneously model the fluctuations of different uncertain parameters with various behaviors for reaching near-reality results as all current methods are only suitable for special uncertain parameters with certain behavior.
4. Integrated energy systems are recognized as one of the effective solutions for increasing the penetration of RESs that offer tremendous benefits, particularly in terms of environmental and economic. However, proposing a comprehensive interconnected structure for MCENs with natural gas, hydrogen, heating and cooling, and electrical energy as well as considering exact mathematical models of different energy networks in the optimization process is a key to gaining confident results. This is while the holistic model is not proposed yet for the integrated structure of EPS, NGG, and DHN to not only simultaneously consider their real states but also include the complete mathematical modeling for them in the coupled modern MCENs.
5. Energy networks with a high/full share of renewables need several reliable ways to improve the system’s flexibility in an unbroken energy supply procedure. Energy management techniques are one of the prevalent flexibility enhancement ways that enable end-users to participate in energy interactions to achieve both technical and economic advantages. However, the related models for demand-side energy management need to be developed in a way to support diverse carriers of energy in MCENs to receive extensive benefits. This is while the comprehensive model is not suggested to simultaneously implement integrated demand-side multi-energy management schemes for increasing the flexibility of EPS, NGG, and DHN in dynamically serving energy altogether.

In order to address the mentioned gaps, this article is aimed to propose a techno-environmental-economic model for optimal scheduling of MCMGs with 100% RESs to facilitate the modernization process of the coupled structure of EPS, NGG, and DHN. To do this, transactive energy technology is intended to create a local multi-vector energy trading market (LMVETM) for developing fair operational models that enable MCMGs for free multi-energy exchange with each other with the aim of dynamically multi-energy balancing in the sharp deregulated environment. Given described research gaps in the previous paragraph, the innovative and distinguishing features of this work are summarized as follows to clearly highlight the superiorities of this work from different aspects in comparison with the previous models.

- The new hybrid structure is proposed for optimal scheduling of MCMGs to allow them to actively participate in MCENs’ interactions with the aim of enabling them to produce 100% clean multi-energy while enhancing their ability to provide continuous multi-energy supply in the sharp deregulated environment. The proposed structure is empowered by different types of RESs to generate carbon-free energy, multi-energy conversion and storage systems to facilitate the renewables’ contribution to energy production, and multi-energy trading and management mechanisms for upsurging the reliability of energy supply. This novel structure supports future modern MCENs to reach the key goal of fully clean energy production in line with energy networks’ decarbonization schemes.
- The transactive energy architecture is developed from uni-vector energy technology to a transactive multi-energy paradigm for structuring LMVETM to prepare free multi-energy sharing possibilities for MCMGs to increase techno-environmental-economic based interoperability among them as well as maximize their chance in time-to-time energy serving in the presence of 100% RESs. To this end, innovative transactive energy-based operational models are proposed for the first time to develop a fair multi-energy exchanging environment for MCMGs, and new mathematical methods are suggested for modeling them in the integrated energy network. In addition to offering a fair economic condition for MCMGs in their multi-energy interactions, the suggested transactive energy model considers the contribution level of each MCMG in the multi-energy sharing of LMVETM to provide appropriate incentives for MCMGs’ contributions to encourage them to balance multi-energy in the local area instead of depending on the upstream network.
- The new hybrid DRCC and SP method is proposed to simultaneously benefit the advantages of both the DRCC and SP approaches for suitably modeling the stochastic behaviors of various uncertain parameters with diverse variation patterns aiming to elicit more realistic results. This innovative uncertainty quantification method increases the ability of the system in the realistic modeling of uncertainties to provide a robust framework for MCMGs to have purposeful energy interactions against the unfavorable variations of RESs.
- The holistic techno-environmental-economic model is offered for the coordinated operation of the EPS, NGG, and DHN to realistically model their interactions by using the complete and exact network modeling for them. The proposed model covers the interconnected structure of the EPS, NGG, and DHN with five energy carriers including hydrogen, natural gas, heating and cooling, and electrical energy that allows MCMGs to benefit from multi-energy interoperability for simultaneously reaching technical, environmental, and economic goals.
- The integrated demand-side multi-energy management schemes (IDSMEM) are developed considering the curtailable and shiftable attributes of elastic demands to increase the system’s flexibility in managing and serving unbroken energy. The proposed IDSMEM enables end-users to participate in multi-energy management programs to not only assist in establishing energy balance but also use this opportunity to increase their economic benefits.

## Multi-carrier multi-microgrids architecture

**Figure 1:**
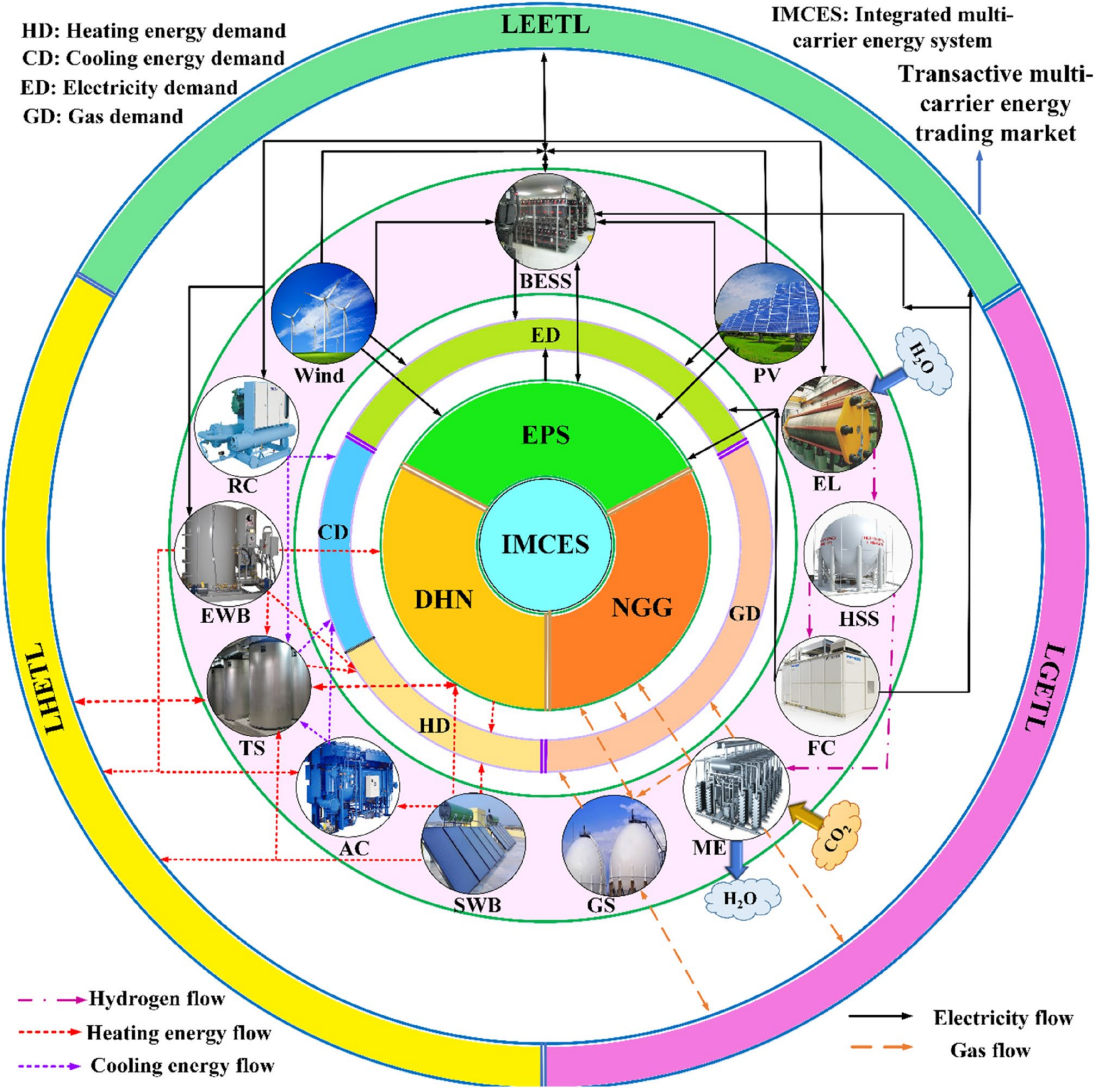
Schematic of the proposed hybrid structure for MCMGs. This structure creates a revolution in energy grids by sustainably integrating several dependent energy networks with multifarious energy carriers. It supports the modernization of future energy grids by enabling the structure to 100% generate clean energy that is realized by developing a novel transactive energy architecture. It also presents the state-of-the-art way of multi-energy interactions that models energy dependences among various energy grids.

The overarching goal of this article is to propose a techno-environmental-economic model for the optimal scheduling of MCMGs targeting to facilitate the modernization process of MCENs. Indeed, in this work, an innovative transactive multi-energy model is proposed for the optimal operation of MCMGs with 100% RESs in modern MCENs. In this respect, as the proposed transactive energy-based model includes gas, heating, cooling, and electrical energy trading in the LMVETM, it is also called the transactive multi-energy exchanging model. In the transactive multi-energy area, hybrid MCMGs refer to those MCMGs that use the multi-energy generation, storage, and conversion units in their energy interactions. In this research, because all MCMGs have the aforementioned features, they are also named hybrid MCMGs. Moreover, the model is proposed for the integrated structure of the EPS, NGG, and DHN, which are called MCENs for short in the transactive multi-energy market. Given that future modern energy grids are targeted to use 100% RESs for multi-energy production, MCMGs are equipped with renewable systems to fully generate unpolluted energy. On the other hand, the architecture of MCMGs is designed in a way to enable them for active participation in the integrated structure of the EPS, NGG, and DHN due to the operation of the future modern MCENs in the interconnected mode. The schematic of MCMGs is depicted in Fig. 1.

Given the proposed structure for MCMGs illustrated in Fig. 1, the system benefits 100% RESs for producing eco-friendly energy. As 100% RESs offer substantial environmental and economic benefits for MCMGs, their uncertain outputs threaten the uninterrupted energy supply. To ensure a continuous supplying multi-energy, the system needs multiple flexibility options to maintain its stability in the presence of stochastic producers. To procure adequate flexibility for the system and support it to be stable against the unfavorable fluctuations of RESs, the proposed structure is empowered by energy storage and conversion units, energy trading strategies, and energy management schemes. Multi-energy storage systems provide the energy storage possibility in energy-rich hours and allow the system to flexibly act by discharging energy when the grid faces energy shortages. Energy conversion units are another way of flexibility and stability improvement that enable MCENs to use the multi-energy conversion process for dynamic meeting energy demand. On the other hand, transactive energy-based energy trading models are proposed to create cooperative energy-sharing possibilities for MCMGs to flexibly manage energy interactions to deliver uninterrupted multi-energy to consumers. Moreover, energy management programs are suggested to control energy on the demand side by effectively utilizing the potential of elastic loads for enhancing flexibility as well as keeping the system’s stability in the unbroken energy serving.

According to Fig. 1, in the EPS part, MCMGs are empowered by solar panels and wind turbines to generate 100% of the electrical energy from renewable systems. Because of the vulnerability of the system under stochastic variations of RESs, the battery energy storage system (BESS) is used to reduce the adverse outcomes of RESs. In the EPS, the excess produced electricity can be used as the entrance energy carrier for the NGG and DHN to support the system for gas and thermal energy generation, respectively. In the DHN part, the effective potential of solar radiation is benefited by using the solar water boiler (SWB) system for clean heating energy production. Moreover, an electric water boiler (EWB) is another employed device for producing heating energy that avails generated electrical energy in the EPS as the entrance energy carrier. For cooling energy, the reciprocating chiller (RC) and absorption chiller (AC) are operated that respectively use the electrical and heating energy for cooling energy generation. Similar to the EPS, thermal energy storage (TS) is deployed for alleviating the risk of RESs usage on the energy production side. On the other hand, the surplus electricity received from the EPS is used for generating natural gas by operating the power to gas (P2G) system in the NGG. In this process, the electrolyzer (EL) system uses electricity for decomposing water into oxygen and hydrogen molars. Afterward, the produced hydrogen molar is stored in the hydrogen storage system (HSS) for later use. At the required time, the stored hydrogen in HSS can be used for natural gas production by the methanization unit (ME) or for electricity generation by the fuel cell (FC). Furthermore, the natural gas storage unit is also intended for upsurging the NGG’s flexibility in uninterrupted serving gas energy load.

In the incorporated structure, the possibility of multi-energy exchange with the main grid is provided for MCMGs to increase their ability not only in uninterrupted meeting multi-energy load but also in maximizing their economic benefits. Herein, as all MCMGs are equipped with 100% RESs for producing multi-energy, a small portion of their energy demand is met by energy trading with the main grid. Indeed, MCMGs can purchase/sell electrical (thermal/gas) energy from/to the EPS (DHN/NGG) to establish a multi-energy balance given their economic and environmental objectives in MCENs. One of the main novelties of this work is to develop the transactive energy architecture from uni-vector energy to the multi-energy technology to structure the LMVETM for MCMGs’ free energy sharing in MCENs. Indeed, all MCMGs can effectively participate in the LMVETM’s interactions by exchanging multi-energy with each other. The LMVETM provides a fair environment for MCMGs to share their surplus energy with the local market not only for increasing their flexibility in balancing energy in the presence of 100% RESs but also for maximizing their economic benefits. According to Fig. 1, energy sharing layers are created for multi-carrier energy, which MCMGs can share energy under the transactive multi-energy paradigm in the local electrical energy trading layer (LEETL) for electricity, the local thermal energy trading layer (LTETL) for thermal energy, and the local gas energy trading layer (LGETL) for gas. The proposed transactive energy architecture provides cooperative energy-sharing opportunities for renewable-based MCMGs in the local area. In the LMVETM, each MCMG can deliver a portion of its surplus energy to the LMVETM in its energy-rich hours of the day for the usage of other MCMGs that require to receive energy for balancing energy. Those MCMGs that received energy from the LMVETM at certain hours of the day should return the energy to the LMVETM with the same economic value that they already received (Models III and IV in the Methods section). This cooperative strategy not only supports all MCMGs to share energy with each other in the LMVETM for dynamically balancing multi-energy in the presence of 100% RESs but also provides a fair economic condition for them according to Eqs. (20) to (23). In other words, these equations mathematically indicate the economic value of the received energy from the LMVETM in some hours of the day needs to be equal to the economic value of the transported energy to the LMVETM in other hours of the same day for all MCMGs.

## Results and discussion

**Figure 2:**
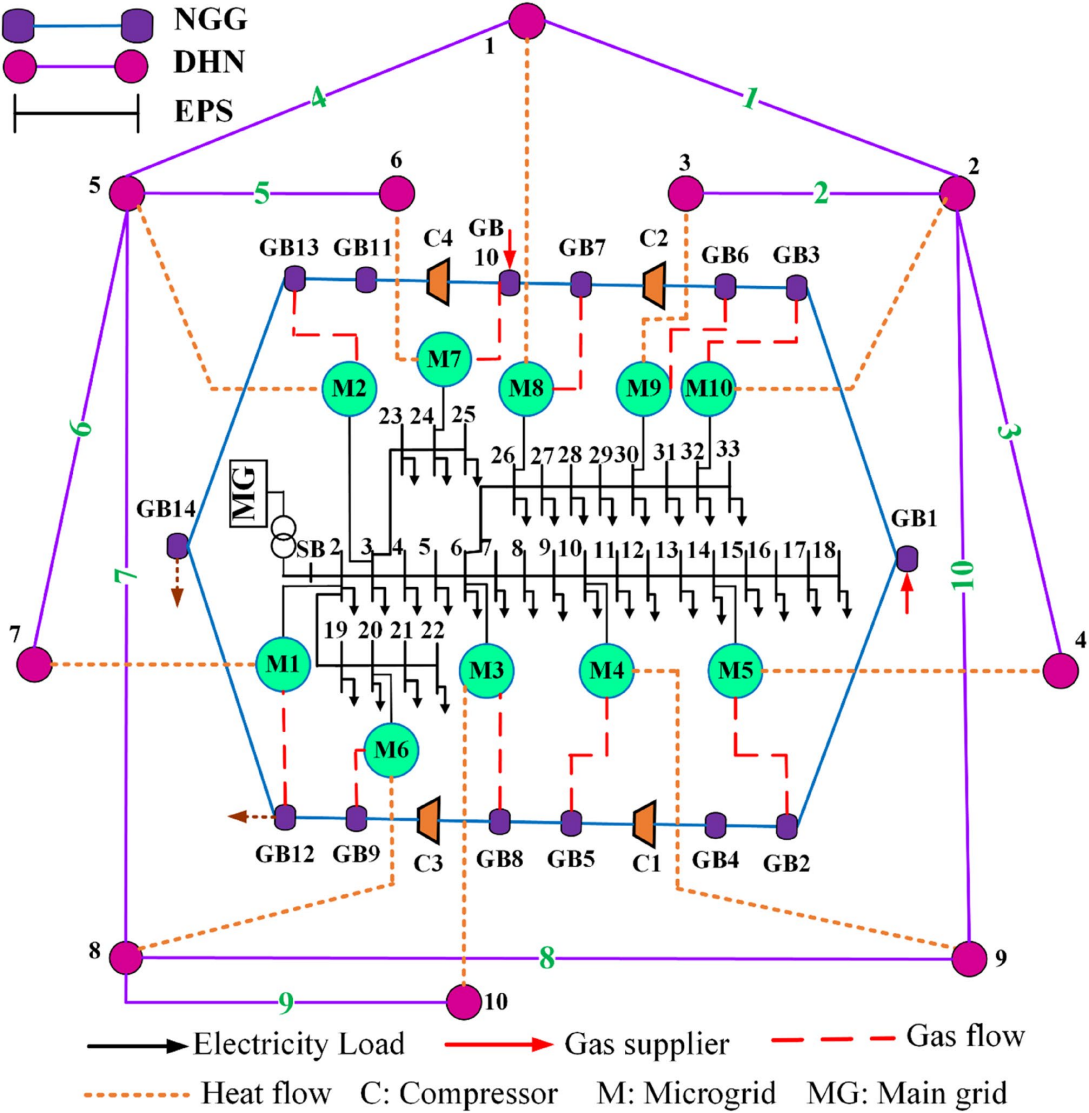
Schematic of the intended test system. The proposed system is the integrated structure of EPS, NGG, and DHN that offers a systematic area of model for facilitating multi-energy interactions in the local area of Chicago.

The overarching goal of this research work is to propose a holistic model for techno-environmental-economic analyzing and optimal scheduling of MCMGs with 100% RESs aiming to facilitate the modernization process of MCENs. The coupled structure of the EPS, NGG, and DHN is considered and the integrated topology of the IEEE 33-bus EPS^22,23^, 14-node NGG^24^, and 10-node DHN^25^ is modified and used as the case study, which its hybrid schematic is portrayed in Fig. 2 for MCMGs in the Chicago area. In this research, it is supposed that energy generation, storage, and conversion systems and technologies are available for the exploitation of MCMGs. Moreover, it is assumed that the distribution grid company manages energy interactions in the hybrid structure and is the owner of MCMGs.

Given Fig. 1, the EPS consists of wind turbines and photovoltaic panels for 100% pollutant-free electricity generation^26^ and a BESS for alleviating the unfavorable outputs of RESs^27^. The DHN comprises the EWB and SWB for heating energy generation, AC and RC for cooling energy production, and TS for upsurging the system’s flexibility in continuous load-serving that their required data are available in^28^. The hydrogen system is deployed that includes the EL for generating hydrogen molar, the HSS for storing generated hydrogen molar, FC unit for using stored hydrogen to produce electricity, and ME in the power to gas (P2G) structure for generating natural gas^29^. The NGG is empowered by the gas storage unit for increasing the assurance of uninterrupted gas supply^24^. For the system assessment, ten MCMGs located in Chicago state are intended that the data related to their electrical, gas, heating, and cooling energy demand and prices can be fully accessed in^30^. The DICOPT and SBB solvers in general algebraic modeling system (GAMS) software^31^ are exerted for solving the scheduling problem. Eliciting the same results from both of them ensures the proper degree of results’ optimality. The total operation costs of MCMGs in Case II (with uncertainty quantification using the proposed hybrid method) and Case I (without uncertainty modeling) are tabulated in Table 1.

**Table 1:** Numerical financial information for the operation of MCMGs in different models.

Studied cases	Total operation cost of MCMGs ($)			
	Model I	Model II	Model III	Model IV
Case I	1,263,166.21	1,144,611.81	1,155,611.03	1,095,995.46
Case II	1,274,742.55	1,194,670.30	1,214,791.42	1,159,235.89

**Figure 3:**
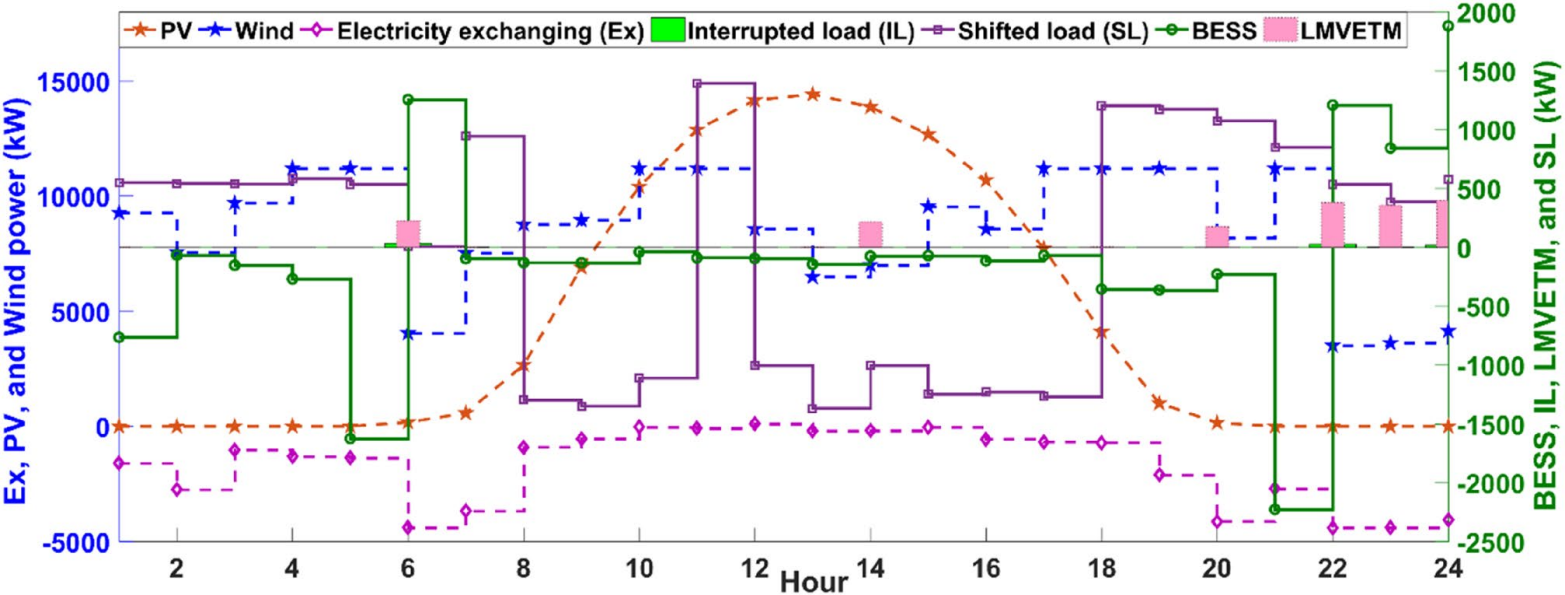
Optimal scheduling of EPS units. It provides a comprehensive overview of how EPS systems are optimally operated in the complex energy structure.

According to Table 1, MCMGs cluster has experienced lower energy costs in Case I than Case II. Indeed, considering the realistic analyzing of the system by modeling stochastic fluctuations for ensuring from sufficient robustness level in Case II is imposed more energy costs for MCMGs in this case than Case I. Additionally, Model IV has the lowest operation costs for MCMGs in comparison with the previous ones. This is because this model not only provides a fair environment for MCMGs’ freely energy exchanging in the LMVETM at a certain level of energy sharing but also procures regulated incentives and costs for motivating MCMGs to mostly use the LMVETM’s potential for dynamically balancing energy instead of highly depending on the main grid with fossil-fuel units. In addition to the reduction of MCMGs’ dependency on the upstream network, participating in the LMVETM’s interactions has been led to a decline in the usage of the other costly ways for balancing energy. In this respect, the optimal operation of the EPS systems is indicated in Fig. 3.

**Figure 4:**
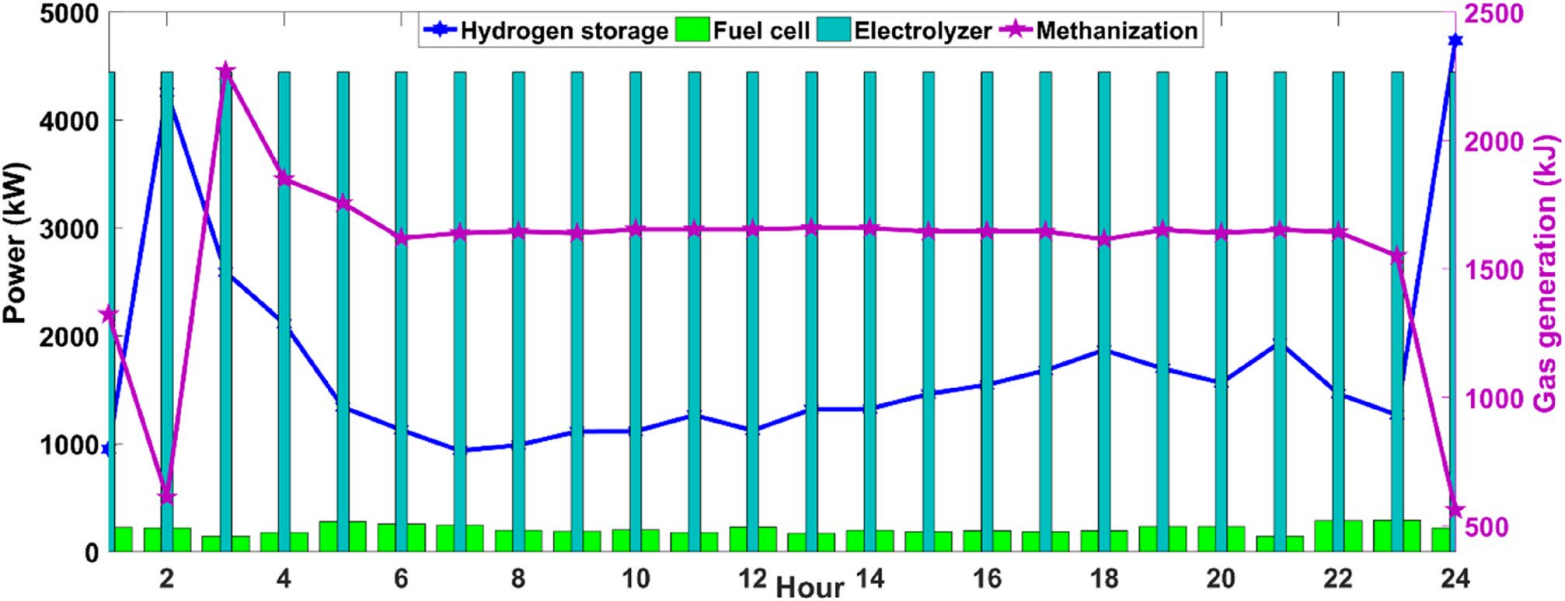
Optimal scheduling of the hydrogen systems. It is a clear view of how energy conversion technology can enhance the capability of operational models in making future energy grids more flexible and sustainable.

As seen in Fig. 3, MCMGs benefit from the unpolluted electricity generation by the wind systems in the early morning and at night while the PV panels are the dominant power production systems in the mid-hours of the day. Due to a lower electricity price from 1 to 8 am, MCMGs have purchased energy from the main grid to serve a portion of the load as well as store energy in BESS for future use. This is while due to the lower energy demand in this period and at night, the excess of produced electricity is contributed in LMVETM’s interactions and shifted load (SL) scheme. However, the favorable PV’ outputs along with the SL program, energy sharing in the LMVETM, and discharging of BESS not only are led to unbroken electricity serving at noon but also a portion of additional energy is sold to the upstream network for revenue maximization. In this work, the hydrogen technology is employed for properly utilizing the surplus electricity production, which the optimal scheduling of the related devices is depicted in Fig. 4.

**Figure 5:**
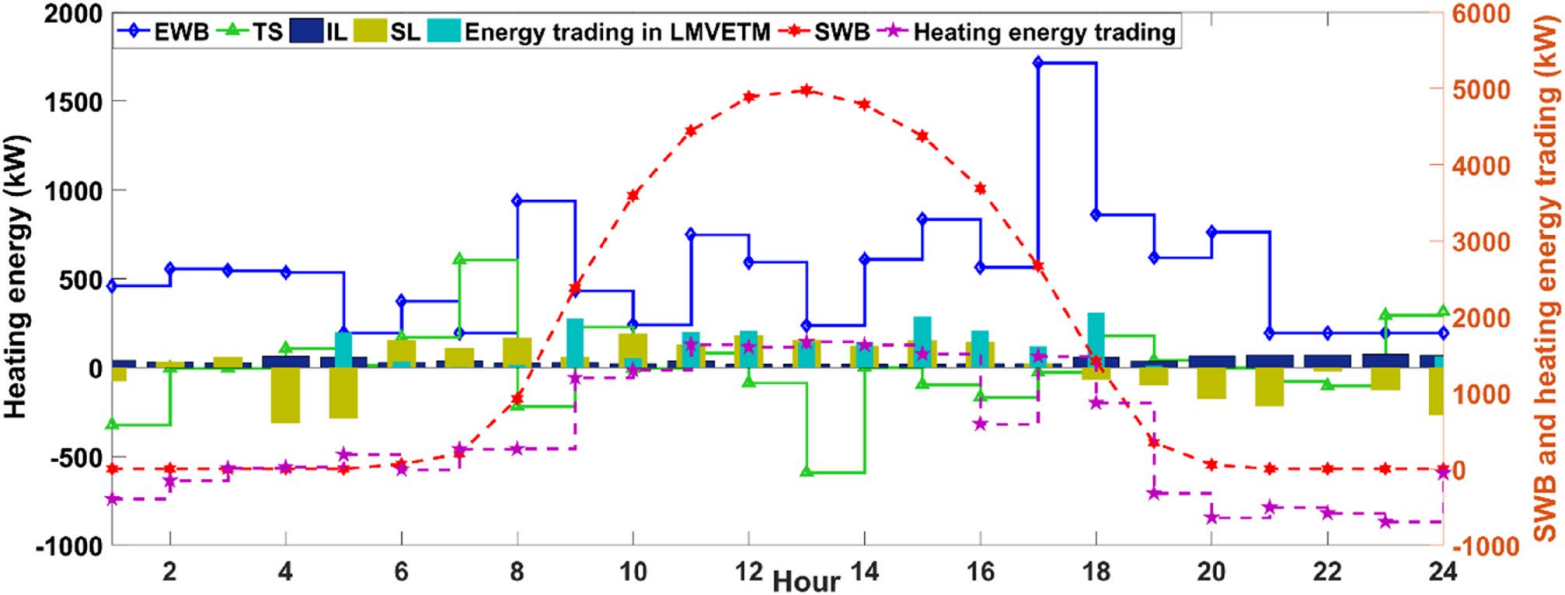
Optimal scheduling of the DHN systems. It is a depth of indication for the operation of DHN systems in the modern structure of multi-energy networks.

To increase synergies among the EPS and NGG, the hydrogen-based systems are exploited to enable MCMGs for possible energy conversion when the clean electricity generation exceeds its consumption. In Fig. 4, the level of stored hydrogen in HSS varies in accordance with the changes in the outputs of FC and ME. For example, when the ME production is dropped from 1 to 2 am, the system witnessed an increment in the hydrogen level of HSS as the EL’s hydrogen generation is almost constant. After 3 am in the early morning, upsurging in the FC production was more prevalent than decreasing the ME’s output that is resulted in declining the HSS’s charge level. However, after 12 am, the system faces approximately constant output of the FC and a slight drop in the ME’s output leads to an increase in the charge level of HSS with a gentle slope. However, remarkably falling in the ME generation from 11 to 12 pm has resulted in substantially enhancing the stored hydrogen in the HSS. In addition to the usage of the excess energy in the hydrogen system, a part of it is injected into the DHN for heating energy production. Figure 5 shows the optimal scheduling of the DHN units during the day.

**Figure 6:**
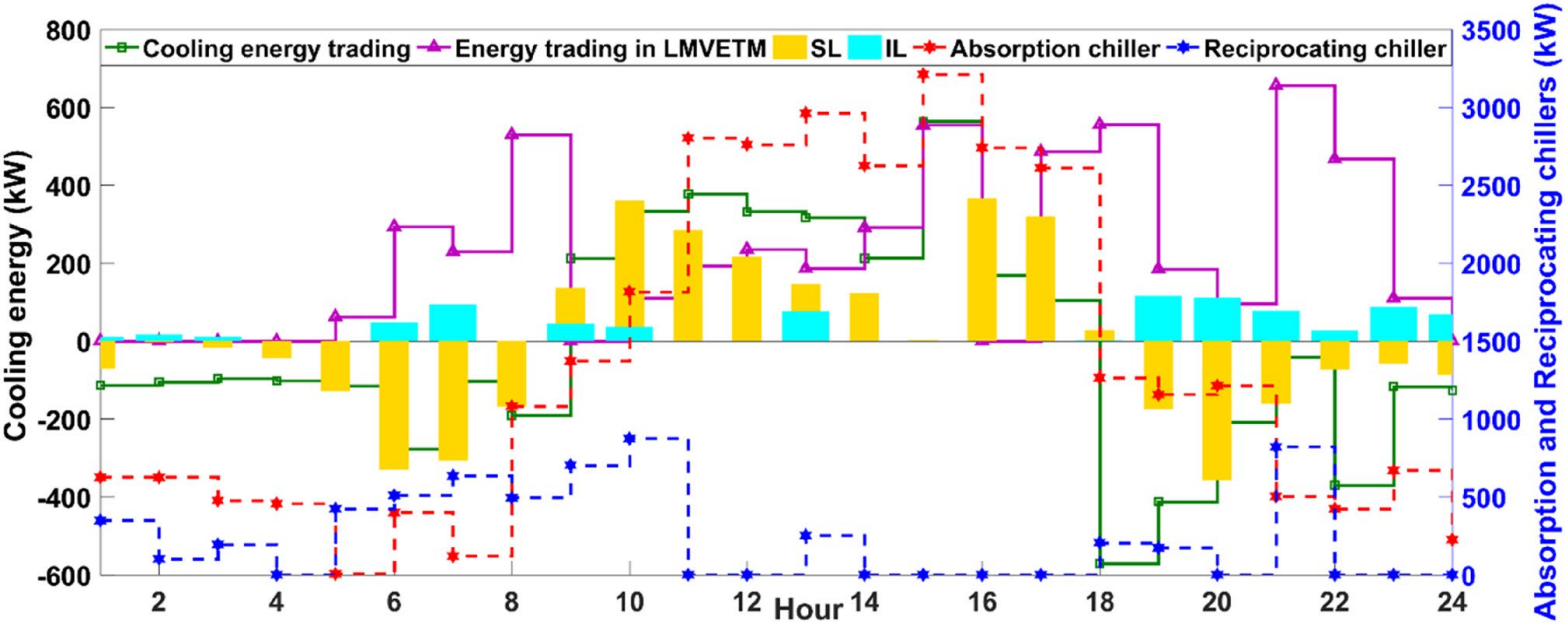
Optimal scheduling of the cooling energy units. It indicates the key role of cooling energy systems in enabling the integrated energy structure for sustainability and balancing multi-energy.

According to Fig. 5, because the heating energy production sector depends on the EWB’s output with limited electricity received from the EPS, the heating energy generation is at the lowest level in the early morning and at night that is led to the use of the SL program for shifting a portion of demand to the mid-hours and serving another portion of demand by purchasing energy from the main grid. This is while by raising the solar irradiation from 8 am, the SWB’s output is significantly increased hour by hour that has enabled MCMGs not only for continuous heating energy supply but also for participating in the LMVETM’s interactions, charging the storage system, and contributing in the demand response schemes. However, when the system has experienced a serious decline in the SWB’s output after 6 pm, the system is empowered by the capability of demand response programs, the EWB unit, and the storage system for dynamically balancing heating energy. In addition to the heating energy, MCMGs are structured aiming to supply the consumers’ required cooling energy in 24-h. The optimal operation of the cooling energy systems is demonstrated in Fig. 6.

**Figure 7:**
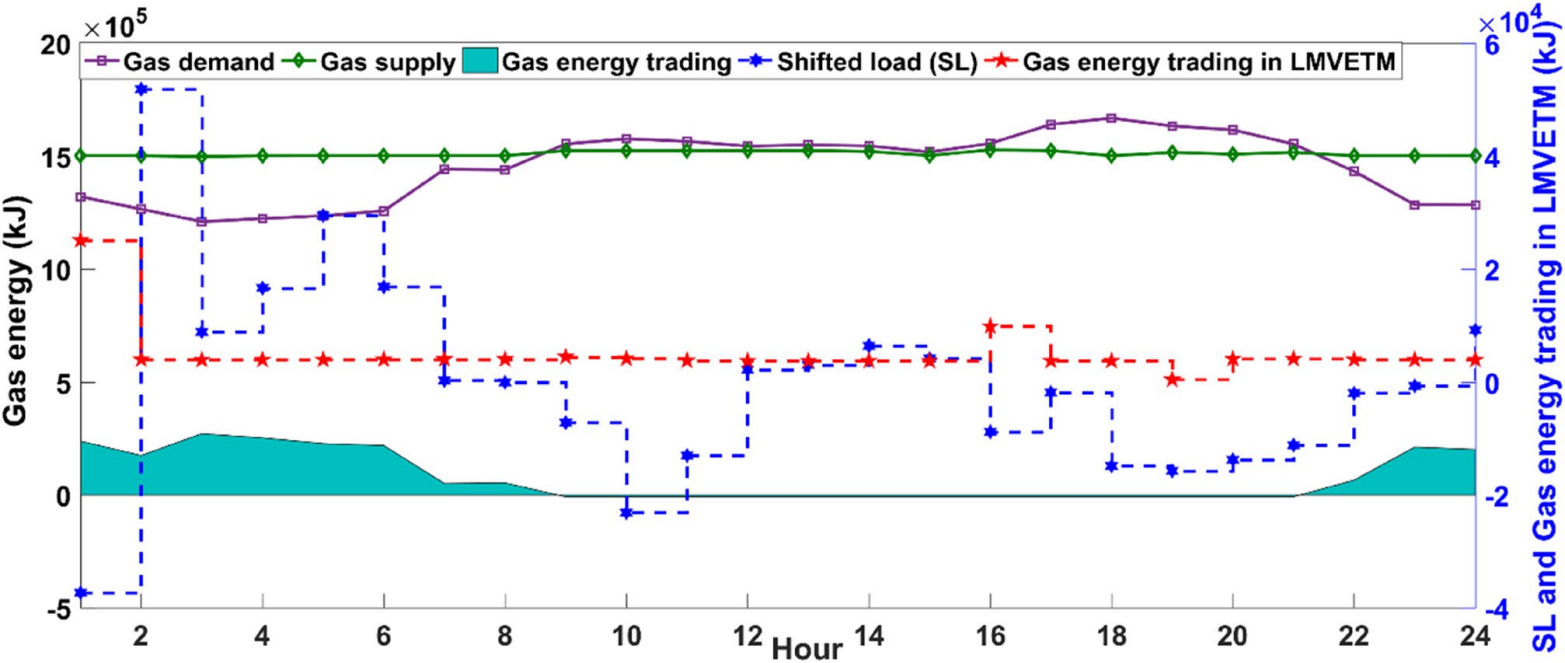
Optimal scheduling of the NGG systems. It specifies how NGG units interact with other parts of the incorporated energy system for maintaining the stability of the grid.

As apparent from Fig. 6, MCMGs have effectively availed the opportunity of cheap energy purchasing along with the SL program for supporting the system in balancing cooling energy in the early morning (1 to 8 am) and at night (7 to 12 pm) that the outputs of AC and RC are in a much smaller value ranging. From 7 am, the system is benefited from raising the sunlight through prolifying the SWB’s output and subsequently increment of the AC production for serving uninterrupted cooling energy, maximizing MCMGs’ revenue by selling energy to the upstream grid, and actively participating in the LMVETM’s interactions. However, after 6 pm, the system has mostly used the effective potential of the LMVETM along with the SL and IL programs, receiving energy from the grid, and the RC for balancing cooling energy in line with the considerable reduction of the AC’s output. In this study, the NGG is optimally operated to reliably supply gas energy in the system. Figure 7 shows the optimal scheduling of NGG systems.

As seen in Fig. 7, greater gas supply than demand from 1 to 9 am and 9 to 12 pm has driven the system to effectively utilize the excess gas energy for supplying shifted gas load in the mentioned periods, involving in LMVETM’s interactions, and selling a portion of it to the NGG for financial benefits. This is while, from 9 am to 9 pm, the potential of gas exchanging in the LMVETM along with the SL program is taken into account rather than the main grid for supporting MCMGs in dynamic gas balancing. Moreover, according to Fig. 4, the ME system has also played a key role in creating a gas balance between its supply and load during the day.

## Conclusions

This paper proposed an innovative transactive energy operational model for the optimal scheduling of MCMGs in modern MCENs. The proposed model is novel for its capability in making the usage of 100% RESs possible for fully carbon-free multi-energy production in the interconnected EPS, NGG, and DHN. Indeed, operating MCMGs under the offered transactive energy-based model enabled them to freely trade energy in the LMVETM to effectively pursue their techno-environmental-economic goals in the deregulated environment. The model is empowered by the new hybrid DRCC and SP approach to model different sources of uncertainties to gain the most realistic and confident results. The assessments in the results section indicated the ability of transactive energy-enabled MCMGs to establish dynamic electrical, gas, heating, and cooling energy balances in the integrated hybrid energy structure with 100% RESs. All MCMGs achieved their techno-environmental-economic goals by intending all operational constraints of MCENs (technical goal), producing fully pollutant-free multi-energy (environmental goal), and obtaining 9.1% cost-saving (economic goal). To sum up, the main critical achievement of this work is the holistic model that enables modern energy networks for the reliable integration of 100% RESs in the multi-carrier energy structure. The effectiveness of the suggested model is examined by a techno-environmental-economic analysis of MCMGs located in Chicago, USA. This investigation illustrated the capability of the proposed model in enabling the hybrid energy structure for the sustainable operation of MCMGs in the presence of 100% RESs.

In modern MCENs, water is one of the key substances that plays a critical role in completing the different energy conversion and interaction processes of the hybrid system. As the availability of water is a prominent issue for optimizing MCENs, the coordinated operation of the EPS, NGG, DHN, and water distribution system, along with the role of the water management unit, will be intended in future work. To assess the effectiveness of the proposed model in both day-ahead and real-time balancing markets, the two-stage stochastic scheduling framework will be developed in future work. The main objective of the first stage day-ahead scheduling problem will be the maximization of MCMGs’ profits while the main objective in the second stage real-time scheduling problem will be the minimization of imbalance costs by minimizing energy imbalances between the bid and actual energy. Moreover, the performance of the proposed model will be analyzed considering the privacy of microgrids to assess their operations from the privacy-preserving viewpoint.

## Methods

Due to the exploitation of 100% RESs in MCMGs, the coordinated operation of MCENs needs sustainable technologies for uninterrupted multi-energy supply in the integrated energy structure. In this work, we have developed the transactive energy architecture to construct a sustainable multi-energy trading area for the energy exchanging of MCMGs. Transactive energy is defined by the GridWise Architecture Council (GWAC) as “a set of economic and control mechanisms that allows the dynamic balance of supply and demand across the entire electrical infrastructure using the value as a key operational parameter”^32^. According to this definition, transactive energy models can be designed based on various economic and control mechanisms with the main mission of dynamically balancing energy between supply and demand, particularly in renewable-dominant energy systems. In other words, one of the main goals of proposed transactive energy models is to support MCMGs to dynamically balance multi-energy between supply and demand in the presence of 100% RESs with uncertain outputs. Employing control and economic mechanisms for establishing a time-to-time energy balance has made transactive energy a suitable tool for improving the efficiency and sustainability of MCMGs with 100% RESs in a reliable and continuous energy supply. Hence, this paper develops the transactive energy architecture for building the LMVETM to allow MCMGs for cooperatively multi-energy sharing aiming to increase the reliability of unbroken multi-energy supply, decrease their dependency on the main grid, and maximize their economic benefits. The four models are intended here for indicating the effectiveness of the proposed one (Model IV).

### Model I

In this model, it is assumed that all MCMGs only have one option for energy sharing and it is the main grid. In other words, MCMGs can only exchange a limited amount of multi-energy with the main grid in Model I and there is no LMVETM for energy trading among MCMGs in this model. The mathematical modeling of Model I is given as.

1 $$\begin{aligned} & {\text{Min }}\;OF_{m} \\ & s.t. \\ & {\text{Constraints}}\;{\text{ in}}\;\Xi^{EGH} \\ \end{aligned}$$

2 $$P_{m,t}^{LtM} = P_{m,t}^{MtL} = 0{ , }G_{m,t}^{LtM} = G_{m,t}^{MtL} = 0$$

3 $$H_{m,t}^{LtM} = H_{m,t}^{MtL} = 0{ , }C_{m,t}^{LtM} = C_{m,t}^{MtL} = 0$$

where,$P_{m,t}^{MtL}$ and $P_{m,t}^{LtM}$(($G_{m,t}^{MtL} {\text{and }}G_{m,t}^{LtM}$)/($H_{m,t}^{MtL} {\text{and }}H_{m,t}^{LtM}$) /($C_{m,t}^{MtL} {\text{and}}C_{m,t}^{LtM}$)) denote the electrical (gas/heating/cooling) energy transmitted from MCMGs to the LMVETM and received it from this market, respectively. $\Xi^{EGH}$ is the set of constraints that include the limitations of DHN, NGG, and EPS and their units that are available in^25,27,28^. Moreover, all constraints of the electric power, heating energy, and natural gas flows are provided in Appendix A that is available in the Supplementary Information file. Given (2) and (3), multi-energy sharing among MCMGs in the LMVETM is not considered in Model I.

### Model II

To increase the flexibility of MCMGs in continuously serving energy load in comparison with Model I, Model II is developed in a way to provide free energy sharing among MCMGs by structuring the LMVETM in the integrated energy system. Indeed, all MCMGs can exchange multi-carrier energy with each other in the LMVETM in line with their optimal scheduling. In Model II, new mathematical formulations are proposed for modeling the LMVETM as follows.

$$\begin{aligned} & {\text{Min }}\;OF_{m} \\ & s.t. \\ & {\text{Constraints }}\;{\text{in}}\;\Xi^{EGH} \\ \end{aligned}$$

4 $$\chi E_{m,t}^{Out} + \chi E_{m,t}^{In} \le 1$$

5 $$\chi G_{m,t}^{Out} + \chi G_{m,t}^{In} \le 1$$

6 $$\chi H_{m,t}^{Out} + \chi H_{m,t}^{In} \le 1$$

7 $$\chi C_{m,t}^{Out} + \chi C_{m,t}^{In} \le 1$$

8 $$P_{m,t}^{LtM} \le \Omega^{EE} .\chi E_{m,t}^{In} { , }P_{m,t}^{MtL} \le \Omega^{EE} .\chi E_{m,t}^{Out}$$

9 $$G_{m,t}^{LtM} \le \Omega^{GE} .\chi G_{m,t}^{In} { , }G_{m,t}^{MtL} \le \Omega^{GE} .\chi G_{m,t}^{Out}$$

10 $$H_{m,t}^{LtM} \le \Omega^{HE} .\chi H_{m,t}^{In} { , }H_{m,t}^{MtL} \le \Omega^{HE} .\chi H_{m,t}^{Out}$$

11 $$C_{m,t}^{LtM} \le \Omega^{CE} .\chi C_{m,t}^{In} { , }C_{m,t}^{MtL} \le \Omega^{CE} .\chi C_{m,t}^{Out}$$

12 $$\sum\limits_{m} {P_{m,t}^{MtL} } = \sum\limits_{m} {P_{m,t}^{LtM} } \, \forall t \in 1:N_{T}$$

13 $$\sum\limits_{m} {G_{m,t}^{MtL} = } \sum\limits_{m} {G_{m,t}^{LtM} } \, \forall t \in 1:N_{T}$$

14 $$\sum\limits_{m} {H_{m,t}^{MtL} } = \sum\limits_{m} {H_{m,t}^{LtM} } \, \forall t \in 1:N_{T}$$

15 $$\sum\limits_{m} {C_{m,t}^{MtL} } = \sum\limits_{m} {C_{m,t}^{LtM} } \, \forall t \in 1:N_{T}$$

16 $$\sum\limits_{t} {P_{m,t}^{MtL} } = \sum\limits_{t} {P_{m,t}^{LtM} } \, \forall m \in 1:N_{m}$$

17 $$\sum\limits_{t} {G_{m,t}^{MtL} } = \sum\limits_{t} {G_{m,t}^{LtM} } \, \forall m \in 1:N_{m}$$

18 $$\sum\limits_{t} {H_{m,t}^{MtL} } = \sum\limits_{t} {H_{m,t}^{LtM} } \, \forall m \in 1:N_{m}$$

19 $$\sum\limits_{t} {C_{m,t}^{MtL} } = \sum\limits_{t} {C_{m,t}^{LtM} } \, \forall m \in 1:N_{m}$$

where, $\chi E_{m,t}^{Out}$ and $\chi E_{m,t}^{In}$(($\chi G_{m,t}^{Out} {\text{and }}\chi G_{m,t}^{In}$)/($\chi H_{m,t}^{Out} {\text{and }}\chi H_{m,t}^{In}$)/($\chi C_{m,t}^{Out} {\text{and}}\chi C_{m,t}^{In}$)) denote the state of the electrical (gas/heating/cooling) energy transmitted from MCMGs to the LMVETM and received it from this market, respectively. $\Omega^{EE} ,\Omega^{GE} ,\Omega^{HE} ,$ and $\Omega^{CE}$ are the respective parameters of upper limits for the electricity, gas, heating, and cooling energy trading with the LMVETM. Equations (4)–(7) indicate the electricity, gas, heating, and cooling energy sharing status between MCMGs and the LMVETM. Equations (8)–(11) limit MCMGs’ electrical, gas, heating, and cooling energy exchanging with the LMVETM. Equations (12)–(15) state the amounts of electricity, gas, heating, and cooling energy received by the LMVETM or transmitted from it should be equal at each time. Equations (16)–(19) denote that the delivered electrical, gas, heating, and cooling energy from each MCMG to the LMVETM should be equal to the amount of energy received by the MCMG from the LMVETM on the same day.

### Model III

Although Model II provides the free multi-energy trading possibility for MCMGs in the LMVETM, it cannot build a fair energy sharing environment for MCMGs. Given constraints (16)–(19), all MCMGs receive the same amount of multi-energy from the LMVETM that they already injected into the LMVETM on the same day. In this energy transaction, the quantity is only considered for the amount of received and transmitted energy while the economic value of the transacted energy is not taken into account. This issue can be led to a fair condition only at those times when the multi-energy price is uniform and has the same value at all hours of the day. This is while the multi-energy price varies during a day and has not a uniform pattern. Therefore, the amounts of received and transmitted multi-energy in the LMVETM by each MCMGs, which are equal in size, can not necessarily be equal in economic value. Under these circumstances, some MCMGs may benefit from LMVETM’s transactions and some may lose and Model II can only provide a fair condition when multi-energy prices are constant at all hours. Thus, to create a fair multi-energy exchange environment for MCMGs in all situations, constraints (16)–(19) in Model II are replaced with constraints (20)–(23) in Model III according to the following formulas.

$$\begin{aligned} & {\text{Min }}\;OF_{m} \\ & s.t. \\ & {\text{Constraints }}\;{\text{in}}\;\Xi^{EGH} \\ & {\text{Constraints }}\left( {4} \right){\text{ to }}\left( {{15}} \right) \\ \end{aligned}$$

20 $$\sum\limits_{t} {\rho_{t}^{EET} .P_{m,t}^{MtL} } = \sum\limits_{t} {\rho_{t}^{EET} .P_{m,t}^{LtM} } \, \forall m \in 1:N_{m}$$

21 $$\sum\limits_{t} {\rho_{t}^{GET} .G_{m,t}^{MtL} } = \sum\limits_{t} {\rho_{t}^{GET} .G_{m,t}^{LtM} } \, \forall m \in 1:N_{m}$$

22 $$\sum\limits_{t} {\rho_{t}^{HET} .H_{m,t}^{MtL} } = \sum\limits_{t} {\rho_{t}^{HET} .H_{m,t}^{LtM} } \, \forall m \in 1:N_{m}$$

23 $$\sum\limits_{t} {\rho_{t}^{CET} .C_{m,t}^{MtL} } = \sum\limits_{t} {\rho_{t}^{CET} .C_{m,t}^{LtM} } \, \forall m \in 1:N_{m}$$

Equations (20)–(23) establish the same economic value for the electricity, gas, heating, and cooling energy transacted by MCMGs in the LMVETM.

### Model IV

Although Model III can fill the key gap of Model II in developing a fair multi-energy trading environment for MCMGs in the LMVETM, this model cannot intend the contribution level of MCMGs in LMVETM’s interactions as this level is different for various MCMGs. Considering the contribution level of MCMGs in LMVETM’s interactions enables the decision-maker to adopt appropriate incentives or costs for energy transactions of MCMGs in a variety of energy sharing levels. This can be conducted with the aim of encouraging MCMGs to increase their activations in the LMVETM for decreasing their dependency on the main grid with fossil fuels while pursuing their economic goals. Hence, Model IV is proposed as the developed version of Model III that models the contribution level of MCMGs by considering incentives and costs for MCMGs’ multi-energy sharing in the LMVETM. In other words, Model IV is targeted to distinguish among those groups of MCMGs that are the deliverers of energy to the LMVETM at most times and other groups that are almost always receivers of energy from the LMVETM for making this market live and active. The proposed mathematical model for Model IV is given as:

24 $${\text{Min }}OF_{m}^{IV} = OF_{m} + \left( {\sum\limits_{t = 1}^{{N_{t} }} {\left[ {(Co_{m,t}^{EET} - En_{m,t}^{EET} ) + (Co_{m,t}^{GET} - En_{m,t}^{GET} )} \right.} } \right.\left. {\left. { + (Co_{m,t}^{HET} - En_{m,t}^{HET} ) + (Co_{m,t}^{CET} - En_{m,t}^{CET} )} \right]} \right) \to \wp_{t}^{M4}$$

$$\begin{aligned} & s.t. \\ & {\text{Constraints}}\;{\text{ in}}\;\Xi^{EGH} \\ & {\text{Constraints }}\left( {4} \right){\text{ to }}\left( {{15}} \right){\text{ and }}\left( {{2}0} \right){\text{ to }}\left( {{23}} \right) \\ \end{aligned}$$

25 $$PT_{m,t}^{EET} = P_{m,t}^{EET} + P_{m,t}^{MtL}$$

26 $$UT_{m,t}^{UET} = U_{m,t}^{UET} + U_{m,t}^{MtL}$$

$$\begin{gathered} UT_{m,t}^{UET} \in \left\{ {GT_{m,t}^{GET} ,HT_{m,t}^{HET} ,CT_{m,t}^{CET} } \right\} \hfill \\ U_{m,t}^{UET} \in \left\{ {G_{m,t}^{GET} ,H_{m,t}^{HET} ,C_{m,t}^{CET} } \right\}{, }U_{m,t}^{MtL} \in \left\{ {G_{m,t}^{MtL} ,H_{m,t}^{MtL} ,C_{m,t}^{MtL} } \right\} \hfill \\ \end{gathered}$$

27 $$\begin{aligned} & Co_{m,t}^{EET} = \left\{ \begin{gathered} 0 \quad { 0} \le \left( {\left( {\sum\limits_{t = 1}^{{N_{T} }} {P_{m,t}^{LtM} } /\sum\limits_{t = 1}^{{N_{T} }} {PT_{m,t}^{EET} } } \right) = Cp_{m,t}^{EET} } \right){\mathbf{ < }}{(1/3)} \hfill \\ \sum\limits_{t}^{{N_{T} }} {\vartheta_{t}^{EE,C1} .(P_{m,t}^{LtM} - (PT_{m,t}^{EET} /3)){ (1/3)} \le Cp_{m,t}^{EET} {\mathbf{ < }}{(2/3)}} \hfill \\ \sum\limits_{t}^{{N_{T} }} {\vartheta_{t}^{EE,C2} .(P_{m,t}^{LtM} - (PT_{m,t}^{EET} /3)){ (2/3)} \le Cp_{m,t}^{EET} \le {1}} \hfill \\ \end{gathered} \right. \\ & \vartheta_{t}^{EE,C1} = \left[ {\left( \frac{1}{3} \right)^{2} .\left( {\frac{{\rho_{t}^{E,MP} }}{{\rho_{t}^{E,P} }} - \frac{{\rho_{t}^{E,OP} }}{{\rho_{t}^{E,MP} }}} \right).\rho_{t}^{EET} } \right]{ , }\vartheta_{t}^{EE,C2} = 2\vartheta_{t}^{EE,C1} \\ \end{aligned}$$

28 $$\begin{aligned} & En_{m,t}^{EET} = \left\{ \begin{gathered} 0 \quad { 0} \le \left( {\left( {\sum\limits_{t = 1}^{{N_{T} }} {P_{m,t}^{MtL} } /\sum\limits_{t = 1}^{{N_{T} }} {PT_{m,t}^{EET} } } \right) = Ep_{m,t}^{EET} } \right){\mathbf{ < }}{(1/3)} \hfill \\ \sum\limits_{t}^{{N_{T} }} {\vartheta_{t}^{EE,E1} .(P_{m,t}^{MtL} - (PT_{m,t}^{EET} /3)){ (1/3)} \le Ep_{m,t}^{EET} {\mathbf{ < }}{(2/3)}} \hfill \\ \sum\limits_{t}^{{N_{T} }} {\vartheta_{t}^{EE,E2} .(P_{m,t}^{MtL} - (PT_{m,t}^{EET} /3)){ (2/3)} \le Ep_{m,t}^{EET} \le {1}} \hfill \\ \end{gathered} \right. \\ & \vartheta_{t}^{EE,E1} = \vartheta_{t}^{EE,C1} { , }\vartheta_{t}^{EE,E2} = \vartheta_{t}^{EE,C2} \\ \end{aligned}$$

29 $$\begin{aligned} & Co_{m,t}^{UET} = \left\{ \begin{gathered} 0 \quad { 0} \le \left( {\left( {\sum\limits_{t = 1}^{{N_{T} }} {U_{m,t}^{LtM} } /\sum\limits_{t = 1}^{{N_{T} }} {UT_{m,t}^{UET} } } \right) = Cp_{m,t}^{UET} } \right){\mathbf{ < }}{(1/3)} \hfill \\ \sum\limits_{t}^{{N_{T} }} {\vartheta_{t}^{UE,C1} .(U_{m,t}^{LtM} - (UT_{m,t}^{UET} /3)){ (1/3)} \le Cp_{m,t}^{UET} {\mathbf{ < }}{(2/3)}} \hfill \\ \sum\limits_{t}^{{N_{T} }} {\vartheta_{t}^{UE,C2} .(U_{m,t}^{LtM} - (UT_{m,t}^{UET} /3)){ (2/3)} \le Cp_{m,t}^{UET} \le {1}} \hfill \\ \end{gathered} \right. \\ & \vartheta_{t}^{UE,C1} = \left[ {\left( \frac{1}{3} \right)^{2} .\left( {\frac{{\rho_{t}^{U,OP} }}{{\rho_{t}^{U,P} }}} \right).\rho_{t}^{UET} } \right]{ , }\vartheta_{t}^{UE,C2} = 2\vartheta_{t}^{UE,C1} \\ & Co_{m,t}^{UET} \in \left\{ {Co_{m,t}^{GET} ,Co_{m,t}^{HET} ,Co_{m,t}^{CET} } \right\}{ , }U_{m,t}^{LtM} \in \left\{ {G_{m,t}^{LtM} ,H_{m,t}^{LtM} ,C_{m,t}^{LtM} } \right\} \\ & \rho_{t}^{UET} \in \left\{ {\rho_{t}^{GET} ,\rho_{t}^{HET} ,\rho_{t}^{CET} } \right\}{ , }Cp_{m,t}^{UET} \in \left\{ {Cp_{m,t}^{GET} ,Cp_{m,t}^{HET} ,Cp_{m,t}^{CET} } \right\} \\ \end{aligned}$$

30 $$\begin{aligned} & En_{m,t}^{UET} = \left\{ \begin{gathered} 0 \quad { 0} \le \left( {\left( {\sum\limits_{t = 1}^{{N_{T} }} {U_{m,t}^{MtL} } /\sum\limits_{t = 1}^{{N_{T} }} {UT_{m,t}^{UET} } } \right) = Ce_{m,t}^{UET} } \right){\mathbf{ < }}{(1/3)} \hfill \\ \sum\limits_{t}^{{N_{T} }} {\vartheta_{t}^{UE,E1} .(U_{m,t}^{MtL} - (UT_{m,t}^{UET} /3)){ (1/3)} \le Ce_{m,t}^{UET} {\mathbf{ < }}{(2/3)}} \hfill \\ \sum\limits_{t}^{{N_{T} }} {\vartheta_{t}^{UE,E2} .(U_{m,t}^{MtL} - (UT_{m,t}^{UET} /3)){ (2/3)} \le Ce_{m,t}^{UET} \le {1}} \hfill \\ \end{gathered} \right. \\ & \vartheta_{t}^{UE,E1} = \vartheta_{t}^{UE,C1} { , }\vartheta_{t}^{UE,E2} = \vartheta_{t}^{UE,C2} \\ & En_{m,t}^{UET} \in \left\{ {En_{m,t}^{GET} ,En_{m,t}^{HET} ,En_{m,t}^{CET} } \right\}{ , }U_{m,t}^{MtL} \in \left\{ {G_{m,t}^{MtL} ,H_{m,t}^{MtL} ,C_{m,t}^{MtL} } \right\} \\ & \rho_{t}^{UET} \in \left\{ {\rho_{t}^{GET} ,\rho_{t}^{HET} ,\rho_{t}^{CET} } \right\}{ , }Ce_{m,t}^{UET} \in \left\{ {Ce_{m,t}^{GET} ,Ce_{m,t}^{HET} ,Ce_{m,t}^{CET} } \right\} \\ \end{aligned}$$

where, $$Co_{m,t}^{EET} ,Co_{m,t}^{GET} ,Co_{m,t}^{HET} ,{\text{and }}Co_{m,t}^{CET}$$ ($$En_{m,t}^{EET} ,En_{m,t}^{GET} ,En_{m,t}^{HET} ,$$ and $$En_{m,t}^{CET} )$$ are the respective indicators of energy transacted cost (incentive) considered for MCMGs’ interactions in the LMVETM. $$PT_{m,t}^{EET}$$($$UT_{m,t}^{UET}$$) denotes the total amount of MCMGs’ dedicated electrical (gas, heating, and cooling) energy for exchanging with the main grid and in the LMVETM. $$\rho_{t}^{E,OP} ,\rho_{t}^{E,MP} ,{\text{and }}\rho_{t}^{E,P}$$ indicate the electrical energy prices at off-peak, mid-peak, and peak times. $$\rho_{t}^{U,OP}$$ and $$\rho_{t}^{U,P}$$ are the representatives for the gas, heating, and cooling energy prices in the off-peak and peak hours. In (24), the second to fifth terms respectively present the amounts of costs and incentives of electrical, gas, heating, and cooling energy for MCMGs. Equations (25) and (26) represent the total amounts of MCMGs’ energy trading for electricity and other energy carriers. Equations (27) and (28) ((29) and (30)) model the cost and incentives considered for MCMGs’ electrical (gas, heating, and cooling) energy sharing in the LMVETM.

### Uncertainty quantification

In recent years, energy networks have been faced with substantial growth in energy consumption in their diverse carriers as well as the proliferation of renewable energy sources (RESs) especially in wind and solar types^33^. As the penetration of these intermittent energy sources is raised to 100%, the challenges associated with their unpredictable attributes come to view more than ever before^34^. Indeed, climate-dependent outputs of RESs threaten the sustainability of the fully equipped renewable system in the integrated energy infrastructure^35^. To promote sustainability in the hybrid energy network, an accurate assessment of the system is one of the undeniable and inevitable steps for adopting appropriate decisions in the presence of 100% RESs. Herein, there is an urgent need for developing capable techniques that enable the system to effectively model the uncertainties with the aim of reaching results close to reality. For this aim, this paper develops a novel hybrid DRCC and SP technique for properly capturing the fluctuations of uncertain parameters in the techno-environmental-economic scheduling of MCMGs equipped with 100% RESs. The overarching feature of the proposed uncertainty quantification method stands on considering the variant behaviors of different uncertain parameters in the deregulated environment.

### Stochastic programming (SP) method

The stochastic programming (SP) techniques typically rely on scenario-based processes in the probability modeling of randomized systems^36^. Their applicability in effectively modeling uncertainties has introduced them as an ideal tool for probabilistic analyzing different problems such as stochastic energy management of power bus charging stations in^37^, the optimal placement of virtual inertia in^38^, and the optimal exploitation of the incorporated electric power and hydrogen system in^39^. Due to considering numerous samples from different points of the uncertainty set, the SP method is recognized as a strong tool more suitable for tackling the uncertainty of RESs with stochastic deviations in a large set^40^. Thereby, this work has benefited from applying the SP method in stochastic modeling of the uncertainties associated with RESs. For this aim, the LHS (for scenario generation) and FFS (for scenario reduction) approaches are deployed in the SP process.

### Latin hypercube sampling technique (LHS)

In the SP, one of the reputed techniques for scenario generation is the LHS, which works based on producing stratified scenarios for the uncertain parameters. Indeed, the LHS is adopted to excerpt samples with an even probability to cover the entire scenario space^41^. Unlike the Monte Carlo (MC) simulation method that generates wholly random scenarios, LHS intends an equal-interval segmentation in producing near-random scenarios that pursue a standard uniform^42^. Maximally stratifying each marginal distribution enables LHS to achieve a superior generalization performance by guaranteeing full coverage of the range of weight variables^43^. To cover the entire sample space for uncertainties stemming from wind speed and solar irradiance with a large-scale uncertainty set, the LHS divides the cumulative probability scale (0 to 1) to ${\mathbb{N}}_{\varpi }$ same-length non-overlapping intervals. By choosing a midpoint of each interval,${\mathbb{N}}_{\varpi }$ scenarios are generated and used in the optimization process considering their corresponding probability. The LHS modeling for $\varpi {\text{th}}$ scenario of the wind speed can be computed as follows.

31 $$\omega_{\varpi } = CDF^{ - 1} \left( {\frac{\varpi - 0.5}{{{\mathbb{N}}_{\varpi } }}} \right) = \sqrt {\ln \left( {\left( {1 - \frac{\varpi - 0.5}{{{\mathbb{N}}_{\varpi } }}} \right)^{{ - 2b^{2} }} } \right)}$$

32 $$CDF(\omega ) = \int_{0}^{\omega } {\left( {e^{{ - (\frac{\omega }{\sqrt 2 b})^{2} }} } \right) \cdot \left( {\frac{\omega }{{b^{2} }}} \right)}$$

where, $\omega_{\varpi }$ is the amount of wind speed $\omega$ in scenario $\varpi {\text{th}}{.}$ Eq. (31) models the wind speed in different scenarios. Equation (32) formulates the cumulative distribution function (CDF) for the wind speed.

### Fast forward selection technique (FFS)

Albeit scenario generation methods provide a beneficent vision by adopting samples from almost all parts of the uncertainty set, the large number of them promotes tremendous challenges for the system. Indeed, high computation workload, complexity, and time-consuming are some crucial challenges that the system faces them in the presence of multitudinous scenarios. To overcome the aforementioned challenges, different scenario reduction methods are offered that one of the effective of them is the fast forward selection (FFS) technique. Generally, the overarching essence of scenario reduction algorithms lies in detecting a new scenario category to maintain the solution close to that obtained by primary scenarios while consists of a fewer number of scenarios in comparison with the original scenario set^40^. For this aim, the FFS concentrates on the scenarios’ Kantorovich distance in computing their distances with each other to sequential select those scenarios that have the shortest distance^6^. Detailed information regarding the process of the FFS approach can be found in^44^.

### Distributionally robust chance-constrained (DRCC) method

The DRCC method intends the embedded underlying true distribution in the ambiguity set for optimizing all goals of probability distributions in the same set^45^. This approach does not need precise probability distributions of uncertain parameters unlike the chance-constrained programming (CCP) and SP^46^. In the DRCC, the probabilistic deviations of the constraints with uncertain parameters are modeled to not only sufficiently raise the robustness of the system but also ensure the achievement of the appointed economic benefit with a certain probability^47^. Indeed, the confidence level of the DRCC method is determined considering the decision maker’s risk preferences^48^. Mathematically formulating the DRCC technique starts by assuming the following distribution for the vector with random variables^47^.

33 $$\Phi \left( {\Theta ,\sigma^{2} } \right) = \left\{ {\Upsilon :E_{\Upsilon } \left[ {\tilde{\Theta }} \right] = \Theta ,Var_{\Upsilon } \left[ {\tilde{\Theta }} \right] = \sigma^{2} } \right\}$$

In (33), all distributions with mean $\Theta$ and variance $\sigma^{2}$ belong to the family of $\Phi .$ According to the family of $\Phi \left( {\Theta ,\sigma^{2} } \right),$ the DRCC for any $\Gamma \in (0,1)$ is modeled as follows.

34 $$\mathop {Inf}\limits_{\Upsilon \in \Phi }^{{}} Prob\left\{ {\tilde{\Theta }^{T} q \le 0} \right\} \ge \Gamma$$

Equation (34) can be equivalent to the convex second-order cone constraint as follows.

35 $$\tilde{\Theta }^{T} q + \sqrt {\tfrac{\Gamma }{1 - \Gamma }} \sqrt {q^{T} \sigma^{2} q} \le 0$$

where, $\Gamma$ denotes the probability of satisfying uncertain constraints in the problem. Detailed information regarding the DRCC model can be fully found in^49^.

### Proposed hybrid DRCC and SP method

**Figure 8:**
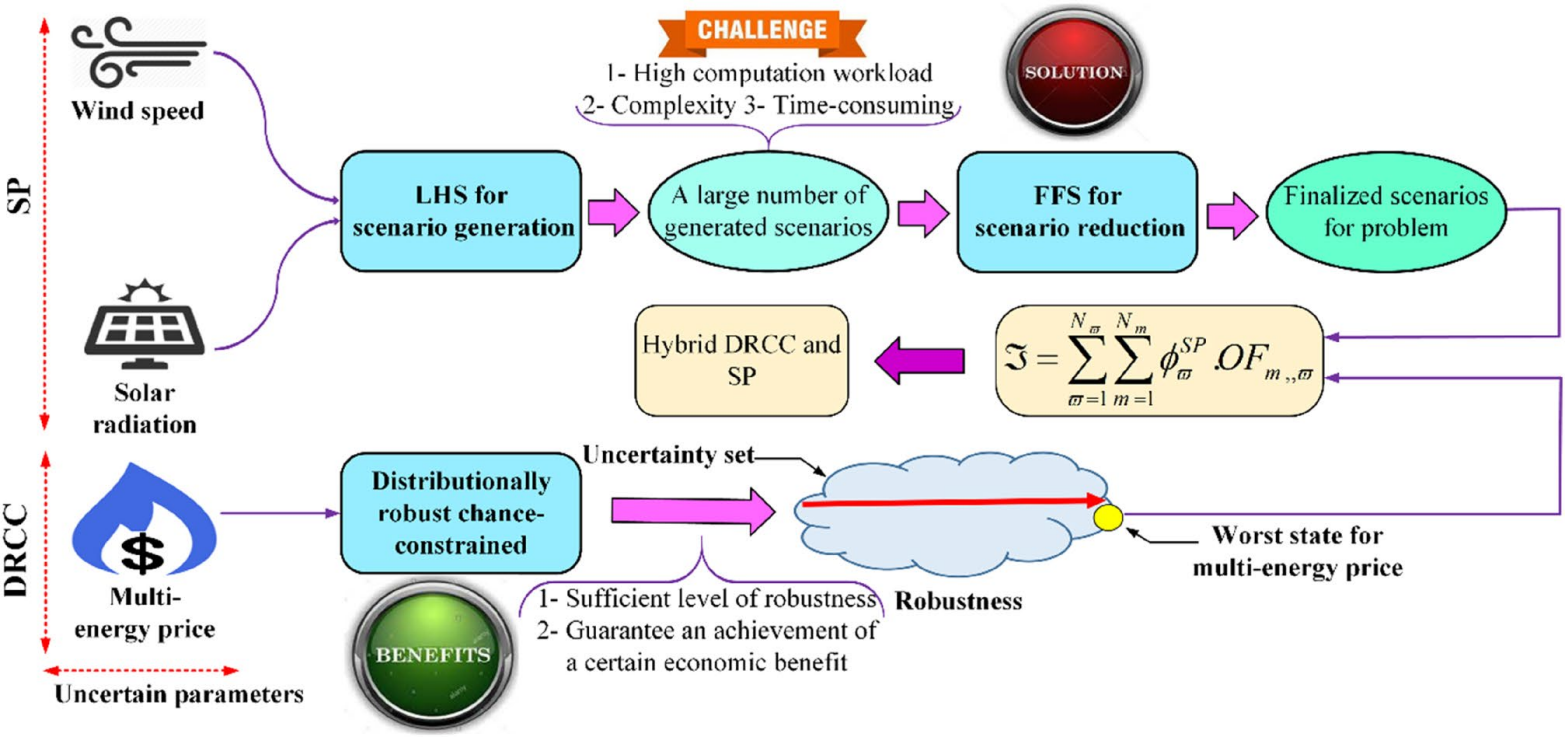
Schematic of the proposed hybrid uncertainty modeling method. It indicates a novel method for modeling uncertainties in energy networks with 100% RESs considering the uncertain behaviors of different uncertain parameters along with the robustness degree of the system.

Today, rapid developments in diverse types of RESs and vehicle electrification, as well as efficient utilization of them, are recognized as the proper opportunities for sustainably developing the modern society threatened by global warming and the energy crisis^50^. Under this circumstance, the system faces a variety of renewable technologies that each of them depends on a certain climate factor in cleaner energy production^51,52^. The climate factors such as solar irradiance and wind speed that are known as uncertain parameters in the energy grid assessments have various behaviors during the day that are different from each other in nature. As considering the different behaviors of uncertain parameters is crucial in accurately modeling MCENs, this prominent issue is completely ignored in recent literature. Therefore, we are motivated to effectively customize the DRCC with the SP method to cope with uncertainties pertaining to wind speed, solar irradiation, and energy prices in techno-environmental-economic optimal scheduling of MCMGs with 100% RESs. Indeed, for the first time, this paper proposes a hybrid DRCC and SP method for effectively utilizing their advantages in suitably tackling the uncertain fluctuations of various uncertain parameters with different variation patterns in the integrated structure of the EPS, NGG, and DHN. The schematic of the suggested hybrid approach is painted in Fig. 8.

The basic mathematical model for the techno-environmental-economic scheduling of MCMGs is considered as Case I for the problem that does not include uncertainty quantification for the scheduling problem. Due to indicate the effectiveness of the offered hybrid uncertainty modeling method, reformulation of the MCMGs’ scheduling problem is carried out here using the mathematical models of the DRCC and SP approaches as Case II. Therefore, we have two cases for the optimization problem: Case II with uncertainty modeling using the proposed hybrid DRCC and SP method while Case I without uncertainty quantification.

### Case I: problem without uncertainty modeling

In this case, the optimal scheduling problem is solved without considering the fluctuating volatilities of uncertain parameters. The mathematical model for Case I is as follows.

$$\begin{aligned} & {\text{Min }}OF_{m}^{IV} \\ & s.t. \\ & {\text{Constraints in}}\;\Xi^{EGH} \\ & {\text{Constraints }}\left( {4} \right){\text{ to }}\left( {{15}} \right), \, \left( {{2}0} \right){\text{ to }}\left( {{23}} \right),{\text{ and }}\left( {{25}} \right){\text{ to }}\left( {{3}0} \right) \\ \end{aligned}$$

### Case II: problem with uncertainty modeling

This case is targeted to assess the optimal scheduling of MCMGs by modeling uncertainties stem from RESs and energy prices using the proposed hybrid DRCC and SP method. For reformulating the problem based on the proposed approach, the scenarios-based objective function can be defined as follows.

36 $$\begin{gathered} OF_{m}^{C2} = \sum\limits_{\varpi = 1}^{{N_{\varpi } }} {\phi_{\varpi }^{SP} .\left[ {\sum\limits_{t = 1}^{{N_{t} }} {\left[ {\left( {\frac{{IC^{BESS} }}{{Ee_{RC}^{BESS} .LCN^{BESS} }}} \right).\left( {P_{m,t}^{BESS} + Ee_{m,t}^{BESS} .\eta^{LL} } \right).\Delta t} \right]} } \right.} \hfill \\ + \sum\limits_{e = 1}^{{N_{e} }} {\sum\limits_{t = 1}^{{N_{t} }} {\left[ {\left( {\rho_{e,t}^{UPR} .(\left| {U_{e,i,t}^{PR} } \right|/2)} \right) + \left( {\gamma_{e,1}^{ULR} .U_{e,i,t}^{LR} + \gamma_{e,2}^{ULR} .(U_{e,i,t}^{LR} )^{2} } \right)} \right]} } + \sum\limits_{t = 1}^{{N_{t} }} {\wp_{t,s}^{M4} } \hfill \\ \left. {\sum\limits_{t = 1}^{{N_{t} }} {\left( {\psi_{m}^{P2G} .\rho_{{CO_{2} }}^{P2G} .\eta^{P2G} .P_{m,t}^{ME} } \right)} - \sum\limits_{e = 1}^{{N_{e} }} {\sum\limits_{t = 1}^{{N_{t} }} {\left[ {\left( {\rho_{e,t}^{UET} .U_{e,m,t}^{UET} } \right).\left( {\rho_{e,t}^{UES} .D_{e,m,t}^{UE} } \right)} \right]} } } \right] \hfill \\ \end{gathered}$$

37 $$\Im = \sum\limits_{m = 1}^{{N_{m} }} {OF_{m}^{C2} }$$

where, $\phi_{\varpi }^{SP}$ states the probability of scenarios. Equation (36) models the scenario-based objective function. In the next step, we use the auxiliary variable ${\mathbb{Z}}$ for facilitating the mathematical implementation of the DRCC concepts:

38 $$\begin{gathered} {\text{Min }}{\mathbb{Z}} \hfill \\ s.t. \hfill \\ \end{gathered}$$

39 $$\begin{aligned} & \mathop {Inf}\limits_{\Re } Pr\left\{ {{\mathbb{Z}} \ge \Im } \right\} \ge \Gamma \\ & \Re = \left\{ \begin{gathered} \rho_{t}^{EET} \in \Phi_{E} { , }\rho_{t}^{GET} \in \Phi_{G} \hfill \\ \rho_{t}^{HET} \in \Phi_{H} { , }\rho_{t}^{CET} \in \Phi_{C} \hfill \\ \end{gathered} \right\} \\ & {\text{Constraints in}}\;\Xi^{EGH} \\ & {\text{Constraints }}\left( {4} \right){\text{ to }}\left( {{15}} \right), \, \left( {{2}0} \right){\text{ to }}\left( {{23}} \right),{\text{ and }}\left( {{25}} \right){\text{ to }}\left( {{3}0} \right) \\ \end{aligned}$$

where, $\Gamma$ denotes the confidence level that is adopted considering the decision maker’s robustness preferences. Eventually, based on the proposed hybrid DRCC and SP method, the final formulation for Case II is as follows.

40 $$\begin{gathered} {\text{Min }}{\mathbb{Z}} \hfill \\ s.t. \hfill \\ {\mathbb{Z}} \ge \Im + \sqrt {\frac{\Gamma }{1 - \Gamma }} .\left( \begin{gathered} \sigma_{{\rho_{t}^{EET} }} .\sqrt {\sum\limits_{t = 1}^{{N_{t} }} {(P_{m,t}^{EET} )^{2} } } + \sigma_{{\rho_{t}^{GET} }} .\sqrt {\sum\limits_{t = 1}^{{N_{t} }} {(P_{m,t}^{GET} )^{2} } } \hfill \\ + \sigma_{{\rho_{t}^{HET} }} .\sqrt {\sum\limits_{t = 1}^{{N_{t} }} {(P_{m,t}^{HET} )^{2} } } + \sigma_{{\rho_{t}^{CET} }} .\sqrt {\sum\limits_{t = 1}^{{N_{t} }} {(P_{m,t}^{CET} )^{2} } } \hfill \\ \end{gathered} \right) \hfill \\ \end{gathered}$$

$$\begin{aligned} & {\text{Constraints in}}\;\Xi^{EGH} \\ & {\text{Constraints }}\left( {4} \right){\text{ to }}\left( {{15}} \right), \, \left( {{2}0} \right){\text{ to }}\left( {{23}} \right),{\text{ and }}\left( {{25}} \right){\text{ to }}\left( {{3}0} \right) \\ \end{aligned}$$

where, $\sigma_{{\rho_{t}^{EET} }} ,\sigma_{{\rho_{t}^{GET} }} ,\sigma_{{\rho_{t}^{HET} }} ,$ and $\sigma_{{\rho_{t}^{CET} }}$ are the respective indicators of standard deviations related to the electricity, gas, heating, and cooling energy trading prices.

## Supplementary Information

Supplementary Information.

## Data Availability

The data relating to this article can be available from Mohammadreza Daneshvar upon reasonable request.

## Code availability

The code cannot be publically shared because of university confidentiality restrictions.
