# Supplementary material for: An innovative transactive energy architecture for community microgrids in modern multi-carrier energy networks: a Chicago case study
Source: Sci Rep. 2023 Jan 27;13:1529. doi: 10.1038/s41598-023-28563-7 (PMC9883243; doi:10.1038/s41598-023-28563-7)
Supplement: Supplementary file 1 — Supplementary Information. [file 41598_2023_28563_MOESM1_ESM.docx]

**Supplementary information**

**TITLE:**

**An innovative transactive energy architecture for community microgrids in modern multi-carrier energy networks- A Chicago case study**

**AUTHORS:**

Mohammadreza Daneshvar^1,*^, Behnam Mohammadi-Ivatloo^1,*^, Kazem Zare^1^

**AUTHOR ADDRESSES:**

^1^Faculty of Electrical and Computer Engineering, University of Tabriz, Tabriz, Iran

**EMAILS:**

Mohammadreza Daneshvar: [m.r.daneshvar@ieee.org](mailto:m.r.daneshvar@ieee.org)

Behnam Mohammadi-Ivatloo: [bmohammadi@tabrizu.ac.ir](mailto:bmohammadi@tabrizu.ac.ir)

Kazem Zare: [kazem.zare@tabrizu.ac.ir](mailto:kazem.zare@tabrizu.ac.ir)

**Correspondence to:** [m.r.daneshvar@ieee.org](mailto:m.r.daneshvar@ieee.org)

**Appendix A:**

In this Appendix, all constraints for the electric power, heating energy, and natural gas flows are provided along with the related descriptions for the mathematical modeling of different energy networks.

1. **Electric power flow modeling**

In the electric power system (EPS) part, the AC power flow constraints are applied to ensure the proposed model is implementable in the practice cases, which are as follows [1].

| $P_{i,t}^{FE}+D_{i,t}^{EE}=P_{i,t}^{GE}\text{ }$ | (1) |
| --- | --- |
| $Q_{i,t}^{FE}+Q_{i,t}^{EE}=Q_{i,t}^{GE}$ | (2) |
| $P_{i,t}^{FE}=\sum_{j=1}^{N_{b}} Y_{i,j}.V_{i,t}.V_{j,t}.\mathrm{Cos}(\theta_{i,j}+\varpi_{j,t}-\varpi_{i,t})$ | (3) |
| $Q_{i,t}^{FE}=-\sum_{j=1}^{N_{b}} Y_{i,j}.V_{i,t}.V_{j,t}.\mathrm{Sin}(\theta_{i,j}+\varpi_{j,t}-\varpi_{i,t})$ | (4) |
|  | (5) |
| $\varpi_{i}^{Min}\leq\varpi_{i,t}\leq\varpi_{i}^{Max}$ | (6) |
| $S_{i,j}^{Min}\leq S_{i,j,t}\leq S_{i,j}^{Max}\text{ }$ | (7) |

where, $P_{i,t}^{GE}$and $Q_{i,t}^{GE}$($P_{i,t}^{FE}$and $Q_{i,t}^{FE}$) are active and reactive power generations (power flows) while their consumptions are indicated by $D_{i,t}^{EE}$and $Q_{i,t}^{EE}$ in line *i* and time *t*. The voltage level (phase angle) is denoted by $V_{i,t}(\varpi_{i,t}).$ The admittance of the line *i-j* (its angle) is stated by $Y_{i,j}(\theta_{i,j}).$ The complex power is also illustrated by $S_{i,j,t}.$Equations (1) and (2) ((3) and (4)) model active and reactive power generations (power flows) in the EPS. Equations (5) to (7) formulate the limitations of voltage, phase angle, and complex power.

1. **Heating energy flow modeling**
2. *Continuity of mass flow*

In the supply and return sides of the district heating network (DHN), the sum of mass flows from different pipelines entering the node is equal to the sum of mass flows leaving the same node [2], as shown below.

| $\sum_{u} (A_{u,h}^{HN,S}m_{u,h,t}^{SP})+(A_{h}^{HS}m_{h,t}^{SN})=\sum_{u} (A_{h,u}^{HN,S}m_{h,u,t}^{SP})+(A_{h}^{HL}m_{h,t}^{LN})$ | (8) |
| --- | --- |
| $\sum_{u} (A_{u,h}^{HN,R}m_{u,h,t}^{RP})+(A_{h}^{HL}m_{h,t}^{LN})=\sum_{u} (A_{h,u}^{HN,R}m_{h,u,t}^{RP})+(A_{h}^{HS}m_{h,t}^{SN})$ | (9) |

where, $m_{u,h,t}^{SP}(m_{h,t}^{SN}\text{ and }m_{h,t}^{LN})$ is the mass flow rate of supply pipelines (heat source and load nodes) in nodes *u* and *h*. $A_{u,h}^{HN,S}(A_{h}^{HS}\text{ and }A_{h}^{HL})$is the incidence matrix for supply pipelines (heat source and load nodes). $m_{u,h,t}^{RP}$($A_{u,h}^{HN,R}$) denotes the mass flow rate (incidence matrix) of return pipelines. Equations (8) and (9) model the mass flow for the heating energy in the supply and return sides of the DHN.

1. *District heating network (DHN) pipeline model*

In the supply and return pipelines, the inevitable heat loss leads to a difference between the temperature of a fluid in the inlet and outlet of the pipeline according to the following formulas [2].

| $T_{h,u,t}^{SP,O}\text{=(}T_{h,u,t}^{SP,I}-T_{t}^{a}\text{)}e^{-\frac{\lambda_{h}^{DHN}L_{h,u}^{DHN}}{\upsilon_{H}^{W}.m_{h,u,t}^{SP}}}+T_{t}^{a}\text{ }\forall h\text{ , }\forall u\text{ ,}\forall t$ | (10) |
| --- | --- |
| $T_{h,u,t}^{RP,O}\text{=(}T_{h,u,t}^{RP,I}-T_{t}^{a}\text{)}e^{-\frac{\lambda_{h}^{DHN}L_{h,u}^{DHN}}{\upsilon_{H}^{W}.m_{h,u,t}^{RP}}}+T_{t}^{a}\text{ }\forall h\text{ , }\forall u\text{ ,}\forall t$ | (11) |

where, $T_{h,u,t}^{SP,O}$and $T_{h,u,t}^{SP,I}$($T_{h,u,t}^{RP,O}$and $T_{h,u,t}^{RP,I}$) are the outlet and inlet temperatures of the supply (return) pipeline. $T_{t}^{a}$and $\upsilon_{H}^{W}$ are the ambient temperature and specific heat capacity for water. $L_{h,u}^{DHN}$and $\lambda_{h}^{DHN}$are the pipeline’s length and heat transfer coefficient. Equations (10) and (11) model the temperature of a fluid in the inlet and outlet of the pipeline.

1. **Natural gas flow modeling**
2. *Gas flow model*

Given the steady-state situation, gas flows can be modeled based on Weymouth’s formula [3]. The amount of gas flow depends on the pressure differences between the inlet and outlet nodes of the pipeline and can be computed as follows [4].

| $G_{g,l,t}^{F,Gas}=Sgn_{g,l}.\varsigma_{Gas}^{F}.\frac{T_{0}^{LP}}{P_{0}^{LP}}.\sqrt{Sgn_{g,l}.\frac{d_{g,l}^{5}.(p_{g,t}^{2}-p_{l,t}^{2})}{T^{LP}.W_{Gas}^{LP}.F_{g,l}^{Di}.G_{Gas}^{F}.L_{g,l}^{Line}}}$ | (12) |
| --- | --- |
| $Sgn_{g,l}=\left\{\begin{matrix} +1\text{ if p}_{g}-p_{l}>0 \\ -1\text{ if p}_{g}-p_{l}<0 \end{matrix}\right.$ | (13) |
| $\varsigma_{Gas}^{F}=\sqrt{\zeta_{Air}^{Gas,F}.\pi^{2}/64}\text{ }$ | (14) |
| $F_{g,l}^{Di}=\frac{0.032}{d_{g,l}^{(1/3)}}$ | (15) |
| $G_{Gas}^{F}=\frac{\zeta_{Air}^{Gas,F}}{C_{Gas}^{LP}}=\frac{1}{0.6}$ | (16) |

where, $G_{g,l,t}^{F,Gas}$is the gas flow in the gas pipeline *g-l* at time *t*. $P_{0}^{LP}$and $T_{0}^{LP}$are initial amounts of the gas pressure and temperature in pipelines. $L_{g,l}^{Line}$and $d_{g,l}$present the length and diameter of the gas pipeline. $W_{Gas}^{LP}$and $C_{Gas}^{LP}$represent the gas compressibility factor and gas constant. $G_{Gas}^{F}$and $\zeta_{Air}^{Gas,F}$state the specific gravity ratio and air constant. $p_{g,t}$is the gas pressure in node *g*. Equations (12) and (13) model the gas flow and its direction in the gas pipeline. Equations (14) and (15) formulate the air constant and the dimensionless friction factor.

1. *Gas network model*

In the natural gas network (NGG), the nodal gas balance should be established at each time to keep the NGG stable [4].

| $G_{g,t}^{SU}+G_{g,t}^{ME}+G_{g,t}^{LtM}+\sum_{l=1}^{N_{l}} (G_{g,l,t}^{F,Gas}-G_{g,l,t}^{Com,Gas})=\sum_{l=1}^{N_{l}} G_{l,g,t}^{F,Gas}+G_{g,t}^{GET}+G_{g,t}^{MtL}+D_{g,t}^{GE}-G_{g,t}^{LR}\text{ }\forall g,t$ | (17) |
| --- | --- |
| $LP_{g,l}^{Min}\leq LP_{g,l,t}\leq LP_{g,l}^{Max}$ | (18) |

where, $G_{g,t}^{SU}$and $G_{g,t}^{ME}$indicate outputs of the gas supplier and methanization unit in the gas node *g* and time *t*. $G_{g,t}^{LtM}$and $G_{g,t}^{MtL}$are gas energy tradings (received and delivered) in the transactive multi-energy trading market. $G_{g,l,t}^{Com,Gas}$and $D_{g,t}^{GE}$denote gas energy consumptions by the compressor and consumers. $G_{g,t}^{GET}$and $G_{g,t}^{LR}$indicate the gas energy trading with the NGG and curtailed gas. $LP_{g,l,t}$states the amount of gas stored in the linepack. Equations (17) and (18) model the gas energy balance and the limitation for the linepack in the NGG.

**References:**

[1] M. Daneshvar, B. Mohammadi-Ivatloo, K. Zare, and S. Asadi, "Two-stage robust stochastic model scheduling for transactive energy based renewable microgrids," *IEEE Transactions on Industrial Informatics,* vol. 16, no. 11, pp. 6857-6867, 2020.

[2] Y. Cao, W. Wei, L. Wu, S. Mei, M. Shahidehpour, and Z. Li, "Decentralized operation of interdependent power distribution network and district heating network: A market-driven approach," *IEEE Transactions on Smart Grid,* vol. 10, no. 5, pp. 5374-5385, 2018.

[3] S. An, Q. Li, and T. W. Gedra, "Natural gas and electricity optimal power flow," in *2003 IEEE PES Transmission and Distribution Conference and Exposition (IEEE Cat. No. 03CH37495)*, 2003, vol. 1, pp. 138-143: IEEE.

[4] J. Qiu, J. Zhao, H. Yang, and Z. Y. Dong, "Optimal scheduling for prosumers in coupled transactive power and gas systems," *IEEE Transactions on Power Systems,* vol. 33, no. 2, pp. 1970-1980, 2017.
